# Oligofructose as an adjunct in treatment of diabetes in NOD mice

**DOI:** 10.1038/srep37627

**Published:** 2016-11-22

**Authors:** Clement Chan, Colin M. Hyslop, Vipul Shrivastava, Andrea Ochoa, Raylene A. Reimer, Carol Huang

**Affiliations:** 1 Department of Biochemistry and Molecular Biology, Alberta Children’s Hospital Research Institute, Cumming School of Medicine, University of Calgary, Calgary, Alberta, Canada; 2 Faculty of Kinesiology, University of Calgary, Calgary, Alberta, Canada; 3 Department of Pediatrics, Alberta Children’s Hospital Research Institute, Cumming School of Medicine, University of Calgary, Calgary, Alberta, Canada

## Abstract

In type 1 diabetes, restoration of normoglycemia can be achieved if the autoimmune attack on beta cells ceases and insulin requirement is met by the residual beta cells. We hypothesize that an adjunctive therapy that reduces insulin demand by increasing insulin sensitivity will improve the efficacy of an immunotherapy in reversing diabetes. We tested the gut microbiota-modulating prebiotic, oligofructose (OFS), as the adjunctive therapy. We treated non-obese diabetic mice with an immunotherapy, monoclonal anti-CD3 antibody (aCD3), with or without concurrent dietary supplement of OFS. After 8 weeks of OFS supplement, the group that received both aCD3 and OFS (aCD3 + OFS) had a higher diabetes remission rate than the group that received aCD3 alone. The aCD3 + OFS group had higher insulin sensitivity accompanied by reduced lymphocytic infiltrate into the pancreatic islets, higher beta-cell proliferation rate, higher pancreatic insulin content, and secreted more insulin in response to glucose. The addition of OFS also caused a change in gut microbiota, with a higher level of *Bifidobacterium* and lower *Clostridium leptum*. Hence, our results suggest that OFS can potentially be an effective therapeutic adjunct in the treatment of type 1 diabetes by improving insulin sensitivity and beta-cell function, leading to improved glycemic control.

Type 1 diabetes (T1D) is an autoimmune disease characterized by the progressive loss of self-tolerance to beta-cell antigens, leading to beta-cell loss^1^. Several immunotherapeutic approaches have been tested for their ability to halt this process^2^,^3^, including the anti-CD3 monoclonal antibody (aCD3). aCD3 has been shown to reverses type 1 diabetes in animal models^4^,^5^ and in humans. It slows down beta-cell loss in patients with newly diagnosed T1D by maintaining some endogenous insulin synthesis capacity in the short term^6^,^7^,^8^. Hence, if an adjunct therapy can improve insulin sensitivity thereby reducing insulin requirement, it may improve the therapeutic potential of aCD3 and future immunotherapeutic approaches. A prebiotic may have a role in this context.

Prebiotics are non-digestible plant-derived carbohydrates such as oligofructose (OFS, an inulin-type fructan) and galacto-oligossacharides^9^,^10^,^11^. Fermentation of OFS produces short-chain fatty acids (SCFA) in the colon, which bind to fatty acid receptors and stimulate glucagon-like peptide-1 (GLP-1) and peptide YY, hormones that reduce appetite, delay gastric emptying, and increase insulin sensitivity^9^,^11^. In a rodent model of insulin-deficient diabetes, OFS improved glycemia, promoted pancreatic insulin production, and increased beta-cell mass^12^. OFS may also improve glycemia through its action on gut microbiota^9^,^13^,^14^,^15^. In both rodent and humans, diabetes is associated with a distinctive gut microbiota^16^,^17^,^18^,^19^,^20^,^21^,^22^,^23^, a higher gram-negative to gram-positive bacterial ratio and a lower bifidobacteria abundance. Bifidobacteria is an important microbial population with many health benefits, and treatment with OFS can dose-dependently increase bifidobacteria and improve glucose tolerance^24^,^25^. Indeed, bifidobacteria level negatively correlates with fasting insulin, and glucose^26^.

In this study, our aim was to determine whether OFS given concomitantly with aCD3 can improve the efficacy of aCD3 therapy in inducing diabetes remission in the non-obese diabetic (NOD) mice, which is a widely used rodent model of type 1 diabetes, and to determine the underlying mechanism. Our results show that indeed, addition of OFS as a therapeutic adjunct improved the efficacy of aCD3 therapy, and is associated with higher insulin sensitivity, higher pancreatic insulin content, and higher insulin secretory response during a glucose tolerance test. Furthermore, the group that received OFS had a lower insulitis score and a higher proportion of insulitis-free islets.

## Results

### The addition of oligofructose increased aCD3-mediated diabetes reversal rate

To investigate whether an oligofructose treatment can improve the efficacy of aCD3 immunotherapy in restoring normoglycemia, diabetic NOD mice were treated with aCD3 alone or aCD3 plus OFS (aCD3 + OFS). Treatment response was assessed 8 weeks after the onset of diabetes, at the completion of the 8-week OFS treatment. We found that most of the mice who responded reverted to normoglycemia within the first 2–3 weeks of treatment. A higher proportion of mice in the aCD3 + OFS group achieved normoglycemia (defined as blood glucose <11 mmol/L) in comparison to the aCD3 group (Table 1): at 8 weeks after the onset of diabetes, 9 out of the 12 mice in the aCD3 + OFS group reverted to normoglycemia while only 4 out of the 15 mice in the aCD3 alone group responded (Fisher’s exact test, p = 0.02).

Blood glucose levels were comparable between the two groups at diabetes onset (13.2 ± 0.8 mmol/L for the aCD3 group and 14.0 ± 0.9 mmol/L for the aCD3 + OFS group). Furthermore, there was no difference in blood glucose levels at diagnosis amongst treated mice that achieved diabetes remission and those that did not respond to treatment (responders: 13.2 ± 0.7 mmol/L, non-responders: 13.7 ± 0.9 mmol/L). These results suggest that OFS can improve the rate of aCD3-mediated diabetes remission.

### Treatment with oligofructose is associated with improved insulin sensitivity

**Figure 1:**
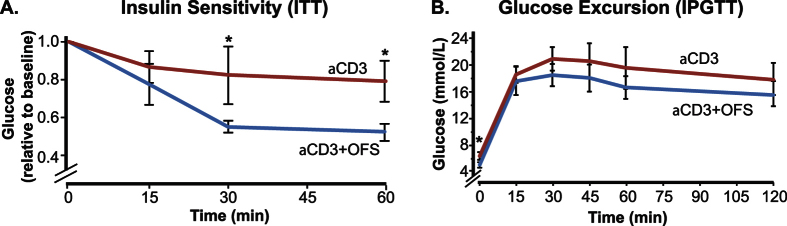
Diabetic NOD mice were treated with aCD3 (5 days) ± OFS (8 weeks). Mice that responded to the treatment and achieved normoglycemia were assessed at the end of the 8-week treatment for insulin sensitivity (**A**), which was measured by a drop in blood glucose in response to intraperitoneal injection of insulin. Glucose tolerance (**B**) was measured by IPGTT. Results are presented as mean ± SEM. n = 4–6 mice/treatment group. *p < 0.05 when comparing the aCD3 to the aCD3 + OFS group at the same time point.

Restoration of normoglycemia can be achieved by an increase in insulin production capacity and/or a reduction in insulin requirement. To determine which of these mechanisms can account for the higher diabetes remission rate in the aCD3 + OFS group, we assessed glucose homeostasis in mice that were in remission after either treatment regimen. At week 8, we found that the aCD3 + OFS group had lower fasting blood glucose and fasting insulin levels (Table 1). The aCD3 + OFS group had higher insulin sensitivity as measured by homeostasis model assessment of insulin resistance (HOMA-IR) and in response to intraperitoneal injection of insulin during an insulin tolerance test (Fig. 1A). Interestingly, there was no significant difference in glucose excursion during an intraperitoneal glucose tolerance test (IPGTT) (Fig. 1B), although the average blood glucose of the aCD3-alone group tended to be higher than the aCD3 + OFS group throughout the IPGTT. The non-diabetic normal control mice and the aCD3-alone group had similar responses during the IPGTT and ITT, as we previously observed^27^. Of note, the aCD3-alone and the aCD3 + OFS groups had similar body weights at the time of sacrifice (aCD3: 23.08 ± 0.43 g vs. aCD3 + OFS: 23.35 ± 0.23 g).

### The addition of oligofructose resulted in a more robust glucose-stimulated insulin secretion

**Figure 2:**
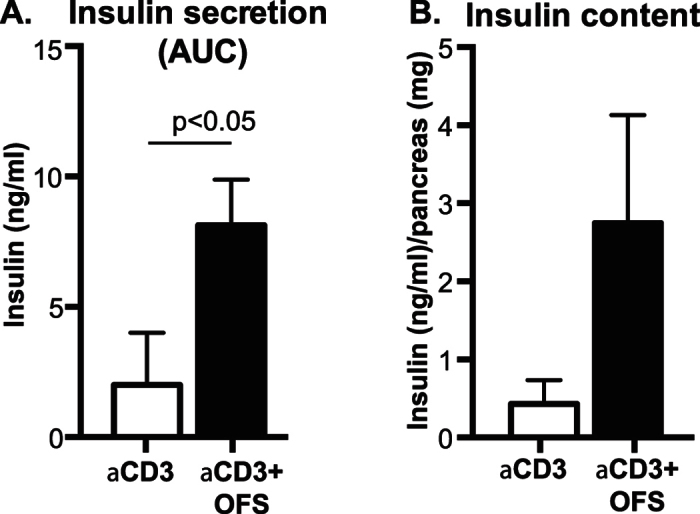
Mice that responded to the treatment and achieved normoglycemia at the end of 8-week treatment were assessed for (**A**) insulin secretion (measured as integrated area under the curve during the IPGTT). Insulin content (**B**) was then assessed in pancreas harvested after 8 weeks of treatment. Results are presented as mean ± SEM. n = 4–6 mice/treatment group. *p < 0.05 when comparing the aCD3 to the aCD3 + OFS group at the same time point.

Next, we determined insulin production capacity by assessing glucose-stimulated insulin secretion and insulin content in mice that responded to treatment and were in diabetes remission. We found that insulin secretion, quantified as integrated area under the curve (AUC) across times 0 to 45 minutes of an IPGTT, was higher in mice treated with aCD3 + OFS in comparison to those treated with aCD3 alone (Fig. 2A). As previously reported and similar to the IPGTT and ITT responses, no difference in insulin secretion was detected between the aCD3-treated group and the non-diabetic control group. Interestingly, several mice in the aCD3 group did not mount a detectable increase in serum insulin level during an IPGTT.

**Figure 3:**
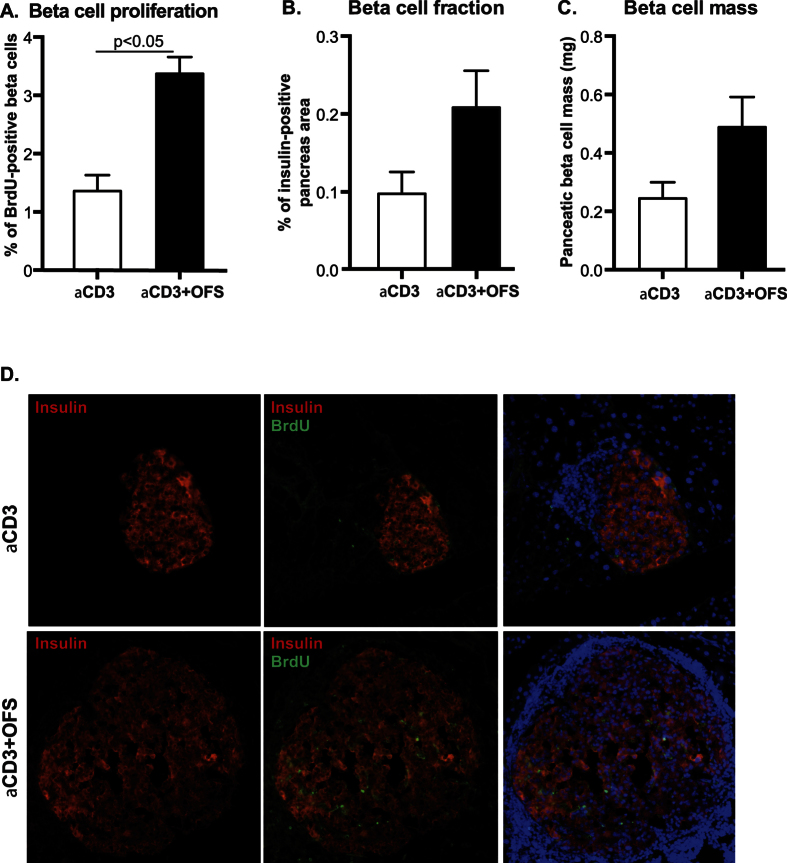
(**A**) Beta-cell proliferation, (**B**) beta-cell fraction, and (**C**) beta-cell mass were compared between the aCD3 and the aCD3 + OFS group at the en d of the 8-week treatment. (**D**) Representative images of the islet, immunostained for insulin (red) and BrdU (green). Results are presented as mean ± SEM. n = 4–6 mice/treatment group. *p < 0.05 when comparing the aCD3 to the aCD3 + OFS group.

The aCD3 + OFS-treated mice also had higher pancreatic insulin content than the aCD3-treated mice (Fig. 2B). This was accompanied by a higher beta-cell proliferation rate and a trend for higher beta-cell fraction as well as beta-cell mass in the aCD3 + OFS group (Fig. 3). Addition of OFS, however, had no impact on beta-cell size, islet size, or islet numbers per pancreas area. It also had no effect on alpha-cell fraction (i.e. fractional area of pancreas positive for glucagon), number, or size (data not shown).

**Figure 4:**
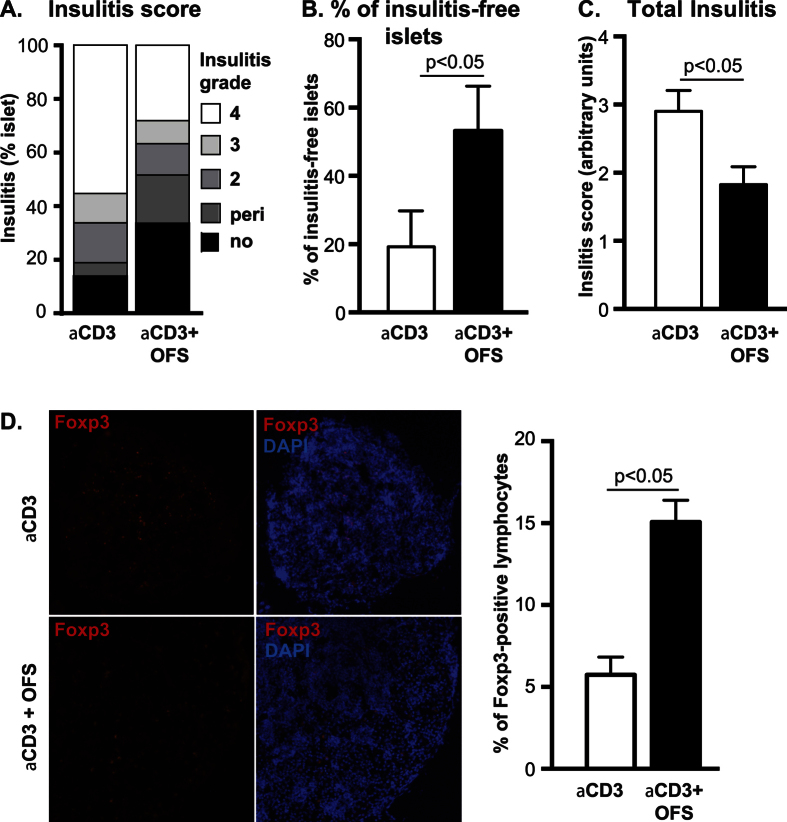
(**A**) Insulitis score, (**B**) percentage of insulitis-free islets, and (**C**) total insulitis score were compared between the aCD3 and the aCD3 + OFS group at the end of the 8-week treatment. (n = 4–6 mice/treatment group, at least 20 islets were counted from each mouse). (**D**) Percentage of Foxp3-positive cells in the pancreatic islets and pancreatic lymph nodes. Representative images of pancreatic lymph nodes, immunostained for Foxp3 (red) are shown. (n = 2–3 mice/treatment group, at least 3500 cells were counted for each group).

Lymphocytic infiltrate of the pancreatic islets, i.e. insulitis, is a hallmark of immune-mediated beta-cell destruction in NOD mice. Here, we found that the aCD3 + OFS-treated mice had a significantly higher proportion of infiltrate-free islets and a lower total insulitis score than mice treated with aCD3 alone (Fig. 4A-C). We also detected a higher number of Foxp3-positive cells in the pancreatic islets and the pancreatic lymph nodes of the aCD3 + OFS group in comparison to the aCD3-alone group (aCD3: 6.04 ± 1.26% vs. aCD3 + OFS: 14.98 ± 1.53%, p < 0.05; >3500 cells counted for each group)(Fig. 4D).

### The composition of gut microbiota changes with oligofructose treatment

We also determined the impact of OFS treatment on the intestinal microbial community, as previous studies in models of type 2 diabetes and obesity suggest that OFS may improve glucose homeostasis by changing the composition of gut microbiota^15^,^28^. Here, we found that total bacteria were not affected by OFS. However, there is a significant decrease in *Clostridium leptum* (p = 0.006) and a significant increase in *Bifidobacterium spp.* (p = 0.04) in those receiving OFS (Table 1).

## Discussion

One of the barriers to successful T1D treatment is the low residual beta-cell mass at the time of diagnosis, which may be insufficient to meet the insulin requirement. The aim of this study was to determine whether a therapeutic adjunct that lowers insulin requirement could improve the efficacy of T1D immunotherapy. We chose OFS because studies have shown that it improves insulin sensitivity in obesity^11^,^15^,^28^,^29^,^30^, which may result in lower insulin requirement and improved glucose homeostasis. Our immunotherapy of choice was the anti-CD3 monoclonal antibody (aCD3) because it is a well-characterized agent that induces permanent diabetes remission in up to 60% of NOD mice^4^,^5^,^27^,^31^, and it has been used in several human trials, demonstrating some efficacy in slowing the process of beta-cell destruction^6^,^7^,^8^,^32^. In this study, we found that when given in conjunction with aCD3, OFS improves the aCD3-mediated diabetes reversal rate in NOD mice, accompanied by an improvement in insulin sensitivity, a reduction in insulitis, and an increase in beta-cell proliferation rate and insulin secretion.

In a rat model of streptozotocin-induced insulin-deficient diabetes, treatment with OFS improved glucose tolerance, promoted pancreatic insulin production, doubled the beta-cell mass, and up regulated GLP-1 levels, which was assumed to be responsible for the positive effects on the beta-cell^12^,^33^. In the NOD mice, we found that OFS improved glucose tolerance, increased pancreatic insulin content, and increased beta-cell proliferation rate which was accompanied by a 2.5-fold increase in beta-cell mass, although the latter did not reach statistical significance. We measured the mRNA expression of proglucagon in the jejunum, the ileum, and the colon. However, we did not detect a difference between the group that received aCD3 + OFS and the group that received aCD3 alone (data not shown). Expression of glucagon in alpha cells is known to be elevated in autoimmune T1D^34^,^35^, but whether the expression of proglucagon in the gut is also elevated is unknown. It is possible that our inability to detect a difference in proglucagon expression between the aCD3 + OFS and the aCD3-alone groups is due to the already up regulated proglucagon levels in the diabetic NOD mouse, such that the addition of OFS cannot increase it further. Interestingly, our data suggest that proglucagon mRNA expression is higher in the diabetic NOD mice than those that have not developed diabetes (data not shown).

OFS may improve glycemia through its action on intestinal mucosal barrier function and gut microbiota^13^,^14^,^15^. Patients with T1D and T2D have distinct gut microbiota in comparison to healthy individuals^18^,^19^,^20^,^21^,^23^,^36^, with a higher gram-negative to gram-positive bacterial ratio and a lower bifidobacteria abundance^16^. Furthermore, diabetes is associated with increased gut permeability, allowing bacterial lipopolysaccharide from the gram-negative bacteria to translocate into the systemic circulation, triggering systemic inflammation and insulin resistance^37^,^38^,^39^,^40^. OFS treatment dose-dependently increases bifidobacteria^24^ and improves glucose tolerance in rodents^25^,^26^. Indeed, bifidobacteria abundance negatively correlates with fasting insulin and glucose levels in mice with high fat diet-induced diabetes^26^. OFS also restores tight junction protein expression in the gut mucosa of obese mice^38^. Here, we found that the OFS-treated NOD mice had a different gut microbiota than those not treated with OFS, with higher levels of *Bifidobacterium* spp. and lower levels of *Clostridium leptum* (cluster IV), which are changes that are associated with improved insulin sensitivity. We also determined the expression level of tight junction molecules zonula occludens-1 (ZO-1) and occludin, but did not detect any difference between the treatment groups (data not shown). Interestingly, the group that received OFS had reduced insulitis, which is consistent with the notion that OFS reduces inflammation. This reduction in insulitis likely contributes to the increase in insulin content, as insulitis causes insulin degranulation.

An interesting observation was that although the aCD3 + OFS group secreted more insulin in response to glucose, we did not detect a significant difference in glucose excursion during the IPGTT between the aCD3-alone and the aCD3 + OFS group. One possible explanation is the relatively wide inter-individual variation in glucose response within each treatment group, such that the overall result did not reach statistical significance, although there was a trend for lower glucose levels in the aCD3 + OFS group than the aCD3 group throughout the test (Fig. 2B).

There were some limitations to this study. First, we only used female NOD mice as <30% of male NOD mice develop diabetes, although our finding should also apply to males since the gut dysbiosis observed in T1D and T2D are present in both sexes^19^,^21^. Second, in regards to the adverse effects of OFS, some of the mice that received OFS developed a significantly distended abdomen, although they were otherwise healthy with normal blood glucose. This may be unique to the autoimmune T1D model due to the highly inflammatory milieu of these mice. Hence, tolerability of OFS will need to be assessed.

In summary, we demonstrated that the use of an insulin sensitizer, in this case OFS, in conjunction with an immune modulatory therapy (i.e. aCD3), is a viable paradigm to induce diabetes remission in early established diabetes. OFS achieves this goal by improving insulin sensitivity and increasing insulin secretory capacity above a threshold required to maintain normoglycemia. Success of this paradigm ultimately depends on the efficacy of immune therapy to halt beta-cell destruction. Dose-finding studies will need to be performed to find a dose of OFS that has a tolerable gastrointestinal side effect profile but still potent enough to induce the change in gut microbiota required to achieve the desired metabolic effect.

## Methods

### Mice

Female 6-week old non-obese diabetic (NOD) mice were purchased from Jackson laboratories. Mice were maintained on 12-h light/dark cycles with *ad libitum* access to food and water. Drinking water was supplemented with 0.8 mg/ml of 5-bromo-2′-deoxyuridine (BrdU) (Sigma-Aldrich) for one week every month to label newly proliferated cells, starting at the onset of diabetes.

### Materials

Anti-CD3 monoclonal antibody (mAb145-2C11, anti-mouseCD3) was purchased from BioXCell (West Lebanon, NH, USA). Oligofructose (Orafti^®^ P95) was provided by Beneo GmbH (Mannheim, Germany) and mixed with normal chow (Laboratory rodent diet 5001, Canada Lab Diet, Leduc, Canada) at 1:9 ratio. Mouse ultrasensitive insulin ELISA kit was from ALPCO (Cat# 80-INSMSU-E01; Salem, NH, USA). All chemicals were purchased from Sigma-Aldrich (St. Louis, MO).

### Ethics

All care and use of animals was approved by the Animal Care Committee at the University of Calgary in accordance with standards of The Canadian Council on Animal Care (Permit number: AC12-0194).

### Treatment

Female NOD mice were screened for hyperglycemia twice weekly starting at 12 weeks of age, and diagnosed with diabetes when serum glucose ≥11 mmol/L on 2 consecutive days. Starting on day 1 of diabetes, experimental mice were treated with aCD3 antibody alone (n = 15) or aCD3 plus oligofructose (OFS) (n = 12), while control mice received either OFS only (n = 3) or no active treatment (n = 5). aCD3 was given intravenously at 10 μg/day for 5 consecutive days, a dosing regimen that leads to diabetes remission in up to 2/3 of the animals within 3 weeks^4^,^41^. For those receiving both aCD3 and OFS, starting on day 1 of diabetes, 10% OFS (Orafti P95, mixed with normal chow at 1:9 ratio) was provided daily in a food cup for ensuing 8 weeks. We chose an 8-week OFS treatment period because previous work in obese rats showed that 6–8 weeks of OFS was sufficient to induce metabolic benefits and a change in gut microbiota^15^,^27^. Blood glucose was monitored twice weekly and diabetes reversal/free was defined as random serum glucose <11 mmol/L for at least 3 consecutive weeks. A group of mice that never developed diabetes were also studied in the same manner as the treated diabetic mice. We were unable to follow the control mice (i.e. mice that received OFS only or no active treatment) long term because they had significant weight loss and became very lethargic, despite of insulin therapy. Consequently, they had to be euthanized ~4 weeks after onset of diabetes.

### Tissue Collection

After the mice were sacrificed by cervical dislocation, the entire intestine was excised and fecal matter collected from the cecum and the colon. The full length of the intestine were flushed with PBS and tissue samples were collected from jejunum, ileum, cecum and proximal colon, snap-frozen and stored at −80 °C.

### RNA extraction and real-time PCR

Total RNA was extracted from jejunum, ileum, and proximal colon using TRIzol reagent (Invitrogen, Carlsbad, USA). Complementary DNA was synthesized using the Quantitect Reverse Transcription Kit (Qiagen). Reactions were carried out in triplicate with QuantiFast SYBR Green Master Mix (Qiagen) at an annealing temperature of 60 °C. Data were collected using the DNA Engine Opticon2 Continuous Fluorescence Detection System and software (Bio-Rad; Philadelphia, PA, USA). The relative amount of RNA was determined by comparison with phosphoglycerate kinase 1 mRNA as a reference gene. The primer sequences used were as follows: proglucagon – CCAAGATCACTGACAAGAAATAGG (forward), TGTACATCCCAA GTGACTGGC (reverse); zonula occuldens-1 (ZO-1) – GAGTTTCGGGTCCGAGGAG (forward), CATTGCTGTGC TCTTAGCGG (reverse); occludin – TGAACTGTGGATTGGCAG CG (forward), AAGATAAG CGAACCTTGGCG (reverse); and phosphoglycerate kinase 1 (PGK1) – CTCCGCTTTCATGTAGAGGAAG (forward), GACATCTCCTAGTTTGGACAGTG (reverse).

### Intraperitoneal glucose tolerance test (IPGTT) and insulin tolerance test (ITT)

IPGTT (glucose at 2 g/kg body weight) and ITT (insulin at 0.5unit/kg body weight) were performed as previously described^42^. Additional blood samples (~30 μl) were taken at times 0, 15, 30 and 45 minutes of IPGTT for insulin concentration measurements by ELISA.

### Pancreatic Insulin Content

Insulin was extracted from the pancreas by incubating the homogenized frozen pancreatic tissue in 1 M HCl/70% ethanol overnight at −20 °C. An equal volume of 1 M TrisHCl (pH = 7.5) was added to the supernatant and stored at −20 °C until insulin determination was carried out by ELISA. Insulin content was normalized to total protein content, which was determined using the BCA Protein Assay Kit (Thermo Scientific, Rockford, Illinois).

### Immunofluorescence

Paraffin-embedded pancreas tissue blocks were sectioned longitudinally to 7 μm, and every 40^th^ section was immunostained for insulin and/or glucagon to identify beta cells as described previously^42^. Nucleus was counter stained by 4′,6-diamidino-2-henylindole (DAPI) to identify cells and to facilitate cell counting. Beta cells were numerated by counting the number of insulin-positive cells by hand. Beta-cell proliferation rate was defined as the percentage of insulin-positive cells that also stained positive for BrdU^42^. Ten tissue sections, ~2500 cells per mouse, were counted (range 543–5972 cells). Pancreas area was defined by nuclear counter staining. Presence of Foxp3-positive regulatory T cells in pancreatic islets and lymph nodes were detected by immunostaining for Foxp3^43^. Antibodies used included: rat anti-BrdU (Abcam) at 1:100, guinea-pig anti-insulin (Dako), rabbit anti-glucagon (Linco) at 1:500, biotinylated anti-mouse/rat Foxp3 (eBioscience) at 1:100, all diluted in 1% goat serum. Antigen retrieval with Target Retrieval Solution (Dako) or 10 mM sodium citrate buffer was used for Foxp3 or BrdU staining, respectively.

### Immunohistochemistry

Paraffin-embedded pancreas sections were immunostained for insulin to identify islets followed by hematoxylin and eosin staining, and degree of lymphocytic infiltration into each islet was scored as following: grade 0 = no infiltrate; grade 1 = peri-islet infiltrate; grade 2 = < 25% of islet area, grade 3 = 25–50% of the islet area, and grade 4 = > 50% of the islet area were infiltrated. At least 20 islets were scored per mouse.

### Islet morphometry

Consecutive images of adjacent non-overlapping areas of the entire pancreas section were acquired using a Leica fluorescence microscope or an Olympus FV1000 scanning confocal fluorescence microscope and captured with a CoolSnap digital camera. Images were analyzed by ImageJ software to measure the insulin- or glucagon-positive area as well as the area of the entire pancreas section^42^. Beta-cell fractions were calculated by dividing the insulin-positive area by the total pancreatic tissue area on the entire section. At least 10 sections were stained and counted for each mouse. Beta-cell mass was determined by multiplying beta-cell fraction by pancreas weight^42^.

### Microbial profiling using qPCR

The contents of the cecum were collected at the time of sacrifice. DNA was extracted from 200 mg cecal matter using the FastDNA Spin kit for feces (MP Biomedicals, LLC, Solon, OH, USA) and quantified using the Nanodrop 2000 (Thermo Fisher Scientific Inc., Waltham, MA, USA). DNA samples were diluted to 4 ng/μl and stored at −20 °C until analysis. Group specific primers, as previously published^15^, were used to profile select microbial groups using the iCycler (Bio-Rad, Hercules, CA, USA). Purified template DNA from reference strains (ATCC, Manassas, VA, USA) was serially diluted to generate a standard curve. Standard curves were normalized to copy number of 16 S rRNA genes using reference strain genome size and 16 S rRNA gene copy number values obtained from the rrnDB^44^. The specificity of the primers and the limit of detection were determined according to Louie *et al*.^45^ Threshold cycle values were used to calculate the number of 16 S rRNA gene copies in each sample.

### Statistical analysis

All statistics were performed using GraphPad Prism VI software (San Diego, CA, USA). Results are expressed as mean ± SEM and comparisons made between the CD3 and CD3 + OFS groups unless otherwise specified. Data was analyzed by two-tailed Student’s *t*-test. Proportions of diabetic mice that reverted to normoglycemia were compared by Fisher’s test. The minimum level of statistical significance was set at p < 0.05.

## Additional Information

**How to cite this article**: Chan, C. *et al*. Oligofructose as an adjunct in treatment of diabetes in NOD mice. *Sci. Rep.* **6**, 37627; doi: 10.1038/srep37627 (2016).

**Publisher’s note:** Springer Nature remains neutral with regard to jurisdictional claims in published maps and institutional affiliations.

## Figures and Tables

**Table 1:** Effect of 8-week OFS supplement on diabetes remission, glucose homeostasis, and fecal microbiota in diabetic NOD mice.

	αCD3	αCD3 + OFS	P value
% of normoglycemic mice	27%	75%	
# of normoglycemic/total # of treated mice	4/15	9/12	χ:^2^ p = 0.02
Fasting glucose (mmol/L)	6.84 ± 0.22	5.05 ± 0.42	0.004
Fasting insulin (ng/ml)	0.88 ± 0.29	0.39 ± 0.06	0.04
HOMA-IR (mmol/L)*(ng/ml)	6.16 ± 2.18	2.52 ± 0.31	0.03
Total Bacteria^#^	7198 ± 1056	7610 ± 1108	0.80
Bacteroides/Prevotella	906 ± 208	1327 ± 346	0.36
Bifidobacterium spp.	41.0 ± 18.4	236 ± 93.9	0.04*
Enterobacteriacea	3.34 ± 0.28	3.17 ± 0.55	0.82
Lactobacillus spp.	440 ± 173	299 ± 74.5	0.43
Clostridium coccoides (Cluster XIV)	2819 ± 216	3341 ± 284	0.19
Clostridium leptum (Cluster IV)	297 ± 46.9	141 ± 27.3	0.006*
Clostridium group (Cluster I)	0.06 ± 0.06	0.15 ± 0.11	0.55
Clostridium group (Cluster XI)	0.07 ± 0.01	0.07 ± 0.01	0.65
Roseburia hominis	0.06 ± 0.06	0.04 ± 0.04	0.86
Methanobrevibacter smithii	1.25 ± 0.19	1.30 ± 0.31	0.90
Akkermansia muciniphila	3.55 ± 1.39	127 ± 90.0	0.26

Comparisons are made between the aCD3 and aCD3 + OFS group by Student’s T tests for all of the above parameters, except for the proportion of mice that achieved diabetes remission, where Fisher’s exact test was performed.

Data for fecal microbiota are presented as 16 S rRNA gene copies (10*3)/20 ng total genomic DNA. All values are presented as mean ± SEM, n = 6 for αCD3 and n = 9 for αCD3 + OFS group.
