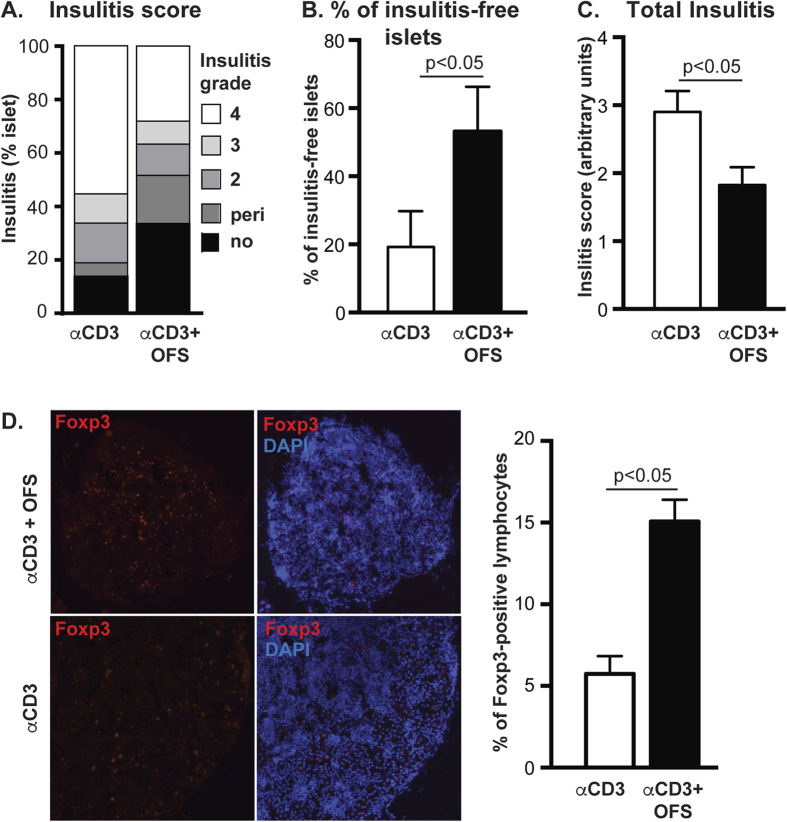# Corrigendum: Oligofructose as an adjunct in treatment of diabetes in NOD mice

**DOI:** 10.1038/srep45800

**Published:** 2017-04-05

**Authors:** Clement Chan, Colin M. Hyslop, Vipul Shrivastava, Andrea Ochoa, Raylene A. Reimer, Carol Huang

Scientific Reports 6: Article number: 3762710.1038/srep37627; published online: 11 22 2016; updated: 04 05 2017

This Article contains an error in the labelling of Figure 4D, where the panel labels ‘αCD3’ and ‘αCD3 + OFS’ are inverted. The correct Figure 4 appears below as Figure 1.

## Figures and Tables

**Figure 1:**